# Neurological signs, symptoms and MRI abnormalities in patients with congenital melanocytic naevi and evaluation of routine MRI-screening: systematic review and meta-analysis

**DOI:** 10.1186/s13023-022-02234-8

**Published:** 2022-03-02

**Authors:** Anne C. Fledderus, Anna Linn Widdershoven, Oren Lapid, Corstiaan C. Breugem, Suzanne G. M. A. Pasmans, Chantal M. A. M. van der Horst, Marc M. Engelen, Phyllis I. Spuls

**Affiliations:** 1 Department of Plastic, Reconstructive and Hand Surgery, Amsterdam University Medical Center, University of Amsterdam, Meibergdreef 9, 1105 AZ Amsterdam, The Netherlands; 2 Department of Dermatology, Amsterdam Public Health, Amsterdam University Medical Center, Meibergdreef 9, 1105 AZ Amsterdam, The Netherlands; 3 Department of Pediatric Neurology, Amsterdam Public Health, Amsterdam University Medical Center, Meibergdreef 9, 1105 AZ Amsterdam, The Netherlands; 4 Department of Dermatology, Erasmus MC University Medical Center Rotterdam-Sophia Children’s Hospital, Doctor Molewaterplein 40, 3015GD Rotterdam, The Netherlands; 5 Department of Plastic, Reconstructive and Hand Surgery, Amsterdam UMC, Location AMC, University of Amsterdam, 1100 DD Amsterdam, The Netherlands

**Keywords:** Congenital melanocytic naevi, Neurocutaneous, MRI, Neuroimaging, Pigment cell, Brain, Central nervous system, Melanoma, Melanocytes, Melanocytosis

## Abstract

**Background:**

A congenital melanocytic naevus (CMN) is a rare skin condition that can be associated with abnormalities of the central nervous system (CNS). These anomalies can sometimes cause severe complications, and rarely death. Adequate information about aetiology and management is therefore crucial. To identify how to monitor patients with CMN, we aimed to estimate the prevalence of neurological involvement in patients with CMN and to summarize what specific neurological signs and symptoms and MRI abnormalities are reported in the medical literature. In addition, we summarized and evaluated the recommendations regarding MRI-screening reported in the medical literature.

**Methods:**

This review was registered in PROSPERO and reported according to the MOOSE checklist. A search was conducted in EMBASE (Ovid), PubMed, and the Cochrane Library. We included studies with 10 or more patients with CMN, reporting on neurological signs and symptoms or CNS MRI. Study selection, data extraction and methodological quality assessment were performed by two independent reviewers. A meta-analysis was used to assess the prevalence of neurological signs and symptoms.

**Results:**

Out of 1287 studies, fourteen studies were eligible for inclusion of which eight were included in the meta-analysis**.** Neurological signs and symptoms prevalence was 7.04% (CI 95% 4.47–10.93%) in the meta-analysis group and 6.26% (95% CI 3.85–10%) in a subgroup of patients with a CMN > 6 cm, evaluated in seven studies. Neurodevelopmental delay and seizures were the most frequently reported signs and symptoms. CNS melanocytosis and hydrocephalus were the most frequently reported MRI abnormalities. It was not possible to estimate the increased risk of neurological involvement in patients with CMN due to low quality of evidence and clinical heterogeneity.

**Conclusion:**

Standardization in CMN studies and a multi-centre prospective study are needed to evaluate neurological involvement. Based on current literature, it is not possible to make strong recommendations on routine MRI-screening. For now, every clinical centre should decide on its own policy and weigh the advantages and disadvantages of routine MRI.

**Supplementary Information:**

The online version contains supplementary material available at 10.1186/s13023-022-02234-8.

## Background

Congenital melanocytic naevi (CMN) are melanocytic skin lesions that sometimes cover large areas of the body. CMN can have a great impact on patients’ lives due to their appearance and risk of development of melanoma or neurological complications [1–3]. The incidence is 1:100 in infants, but large (> 20 cm Projected Adult Size (PAS)) and giant CMN (> 40 cm PAS) are rare and have an incidence of 1:20,000 and 1:50,000 infants, respectively [4]. CMN are caused by a postzygotic mosaic mutation in the embryonic precursor cells of melanocytes in the ectoderm [1, 5]. This mutation can be found anywhere on the skin and/or the central nervous system (CNS).

Various neurological complications are described ranging from mild or no symptoms to death [6, 7]. Some patients with cutaneous CMN can have CNS melanocytic deposits. These deposits are most often benign, as is almost always the case when they are parenchymal [8]. There is no evidence that melanocytic deposits cause CNS pathology. It is only when CNS transformation to melanoma or leptomeningeal melanocytosis occurs that the condition manifests itself, and is often fatal [8]. Infrequently other CNS abnormalities are described as well [7].

Melanin can cause increased signal on T1-weighted images, and occasionally a corresponding decreased signal can also be seen on T2-weighted images [9].

Neurological abnormalities of patients with CMN were traditionally termed ‘neurocutaneous melanosis’ [1]. Suggestions are made to discontinue the use of this term and describe the specific abnormality found [6, 7].

Paediatricians, dermatologists, surgeons and neurologists responsible for the care of patients with CMN, struggle with the management strategies regarding neurological involvement in this patient group due to the rarity of this condition. To inform specialists involved in clinical care of patients with CMN, we aim to estimate the prevalence of neurological involvement (neurological signs and symptoms and MRI abnormalities) in patients with CMN and to summarized what specific neurological signs and symptoms and MRI abnormalities are reported in the medical literature in order to identify what abnormalities should be expected in this patient group. In addition, we summarize and evaluate the recommendations on routine MRI-screening reported in the medical literature.

## Methods

This systematic review was registered in PROSPERO (ID = CRD42020177555) and reported according to the MOOSE checklist [10] and the Joanna Briggs Institute (JBI) guide for prevalence systematic reviews [11].

### Literature search

A systematic search was conducted to find any study that reported CMN and neurological signs and symptoms and/or MRI findings (Additional file 1: S1). An information specialist (FE) was consulted to develop the search strategy and perform the search. The search was performed in PubMed, EMBASE and the Cochrane Library in February 2021. References of included studies were searched for potentially eligible studies.

### Study selection and data extraction

We included all studies without year limits, in Dutch and English, assessing neurological signs and symptoms and CNS MRI abnormalities in ten or more patients. We included studies with patients of any age with CMN > 1.5 cm. We included systematic reviews, cross-sectional studies, cohort studies and controlled clinical trials. Case reports, descriptive reviews and letters to the editor were excluded. When more than one study was published concerning the same patient cohort or case-series, we only included the most recent article with the most detailed description of that particular cohort.

Study selection and data extraction were performed by two independent reviewers to assess eligibility (ACF, ALW) and disagreement was resolved through discussion with another author (ME). The title and abstract of the studies and subsequently the full text of the selected studies were screened. Authors were contacted when articles were not available.

The following data was extracted: study details, patient and CMN characteristics, follow up time, neurological signs and symptoms, CNS MRI characteristics/abnormalities, location of melanocytosis, death due to neurological disease and recommendations for MRI-screening.

### Risk of bias and quality assessment

The risk of bias was assessed by two independent reviewers (ACF, ALW) using the JBI Prevalence Critical Appraisal Tool [11]. Quality assessment was performed with GRADE methodology for quality assessment on the outcome level and the Oxford Centre for Evidence-based Medicine for ratings of individual studies [12, 13]. Studies were not excluded based on their methodological quality as we expected that the study quality would be generally low [14].

### Analysis

We provided a narrative overview of the following outcomes: specific neurological signs and symptoms, MRI abnormalities, deceased patients and recommendations on MRI-screening. In addition to the PROSPERO protocol, we performed a meta-analysis of weighted means of proportions to estimate the prevalence of neurological involvement in R studio version 1.2.1335. We used a random-effects model, as this is advised for prevalence analysis [11]. In contrast to the protocol, we did not exclude studies with a high risk of bias as we wanted to provide an estimate of the best available evidence. We excluded studies with a high risk of selection bias for this analysis. When considerable statistical heterogeneity (I^2^ > 70%) was found, we performed subgroup analysis in groups with similar patient characteristics [15, 16].

## Results

### Search and selection

**Fig. 1:**
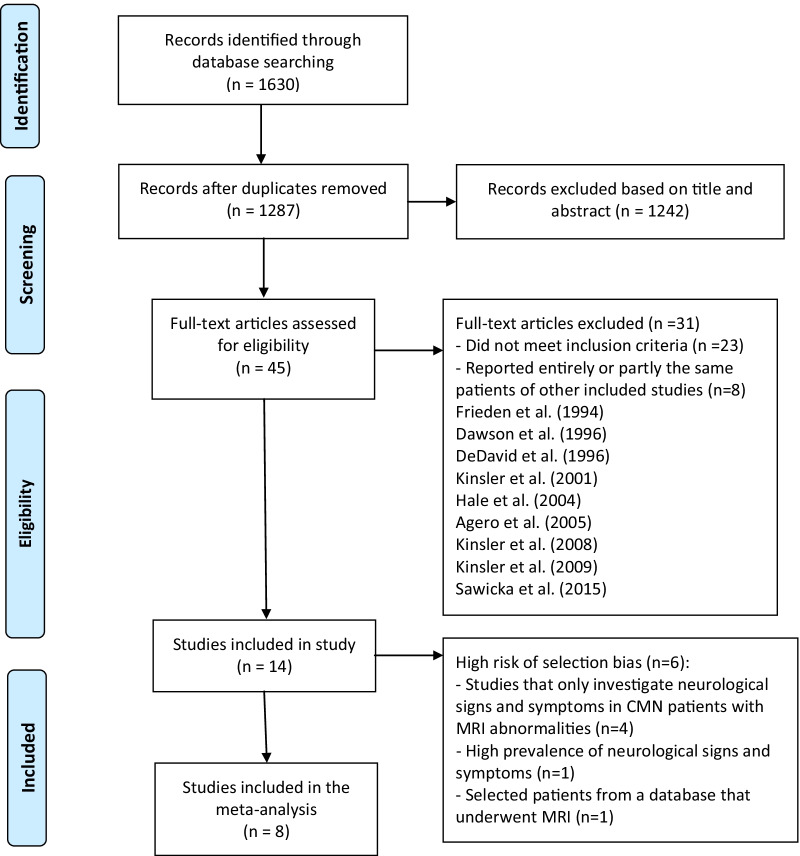
Flow diagram of study selection. CMN, congenital melanocytic naevi, MRI, magnetic resonance imaging

Fourteen studies, reporting on 2339 patients, met the inclusion criteria. The study selection flow diagram is presented in Fig. 1.

### Data extraction and risk of bias assessment

Study, patient and CMN characteristics are shown in Table 1. The mean patient age was 5 years and 6 months and ranged between 1 day and 59 years. The female to male ratio was 1.03:1. The classifications/definitions of different CMN size and number of CMN groups were heterogeneous among studies.

**Table 1:** Study characteristics

References	Country	Patient number^†^	Patient age, mean (range; median)	Female: male ratio	CMN location	CMN size and inclusion criteria	Follow-up time mean (range; median)	Level of evidence^‡^
Ruiz-Maldonado et al. [17]	Mexico	13	11 yr 7 mo (0.6–32 yr)	8:5	Whole body	'Giant' CMN involving the head and neck in reportedly asymptomatic patients	Unreported	4
Bittencourt et al. [23]	U.S.A	160	6 yr 6 mo (1–63 yr; 1.2 yr)	89:71	Whole body	≥ 20 cm or ≥ 20 cm PAS	66.2 mo SD 64.1 (1–238 mo; 42 mo)	3
Foster et al. [9]	U.S.A	46	5 mo (4 days–8 yr)	22:27	Head, neck, trunk	≥ 9 cm (scalp) or ≥ 6 cm (body) CMN involving the head, neck and dorsal spine	5 yr (2–8 yr) (of 44pt)	3
Bett et al. [48]	U.S.A	1072	6 yr 11 mo (1–23 yr)	22:23	Head, neck, trunk (information of extremities CMN available)	≥ 9 cm (scalp), ≥ 6 cm (body) or nevus covering a substantial part of a small body area (face, hand, foot), multiple small/medium	5.6 yr	3
Chan et al. [25]	Singapore	39	18 yr 11 mo (1.9–60 yr)	16:23	Whole body	> 10 cm	16.9 yr (12 mo–38 yr; 15.4 yr)	3
Lovett et al. [20]	Canada	54	1 yr 4 mo (0–14 yr)	29:25	Whole body	≥ 9 cm (scalp) or ≥ 6 cm (body) in infants, ≥ 20 cm PAS or at least 3 medium-sized (1.5–19.9 cm diameter) CMN of the head	89 mo (82.5 mo)	3
Ramaswamy et al. [19]	U.S.A	14	(1–6 yr; 2.6 yr) (of 8 survivors)	3:11	Unreported	High risk patients, according criteria of Kadonaga and Frieden, ≥ 20 cm PAS or multiple small/medium	Unreported	4
Bekiesińska-Figatowska et al. [47]	Poland	24	(12 days–7 yr)	4:3	Whole body	≥ 9 cm (scalp) or ≥ 6 cm (body), ≥ PAS	(15–62 mo)	4
Price et al. [55]	U.S.A	45	Unreported	28:17	Whole body	> 10 cm PAS and multiple CMN > 1, 5 cm PAS, Krengel classifications	No follow up	4
Waelchli et al. [7]	U.K	636	1 yr 6 mo (0.6 yr)	1:2	Whole body	Before 2008: > 2 cm/1% BSA (overlying spine or brain) after 2008: > 1 CMN (regardless the size)	11.0 yr (8.5 yr)	3
Viana et al. [56]	Brazil	57	8 yr 4 mo (2 yr 5 mo)	28:29	Whole body	≥ 20 cm or ≥ 20 cm PAS	5.5 yr ± 3.8 SD, (5.2 yr)	3
Wramp et al. [24]	Germany	83	11 yr 4 mo	41:42	Whole body	> 15–20 cm PAS, Krengel classification	4.6 yr	3
Jakchairoongruang et al. [18]	U.S.A	80	1 yr 10 mo (1 day–22 yr; 6 mo)	Unreported	Unreported	'Large' CMN	(1 mo–11 yr) (of 9pt with brain melanocytosis)	3
Qian et al. [21]	China	13	36 yr 6 mo (18–59 yr)	4:9	Whole body	CMN > 20 cm and/or multiple (3 or more) small/medium CMN (neurocutaneous melanocytosis according Kadonaga and Frieden, included 3 patients without cutaneous CMN)	3 yr	4

CMN, congenital melanocytic naevi; Mo, months; PAS, projected adult size; yr, years

^†^CMN patient included in the study

^‡^According to the Oxford Centre for Evidence-based Medicine

**Fig. 2:**
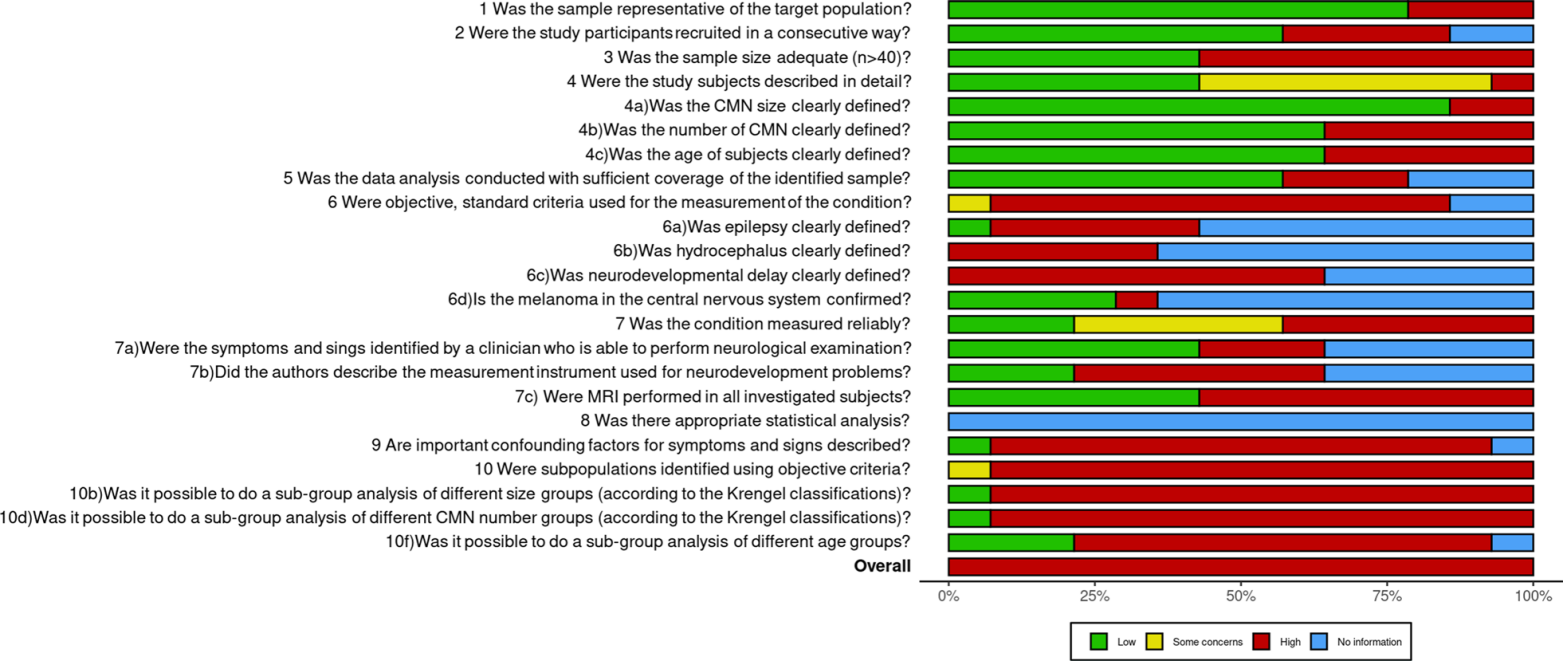
Risk of bias assessment

Figure 2 shows the risk of bias assessment and Additional file 1: S2 shows the complete risk of bias assessment. High risk of bias was found in all studies, regardless of the number of patients included.

### Analysis and quality assessment

**Fig. 3:**
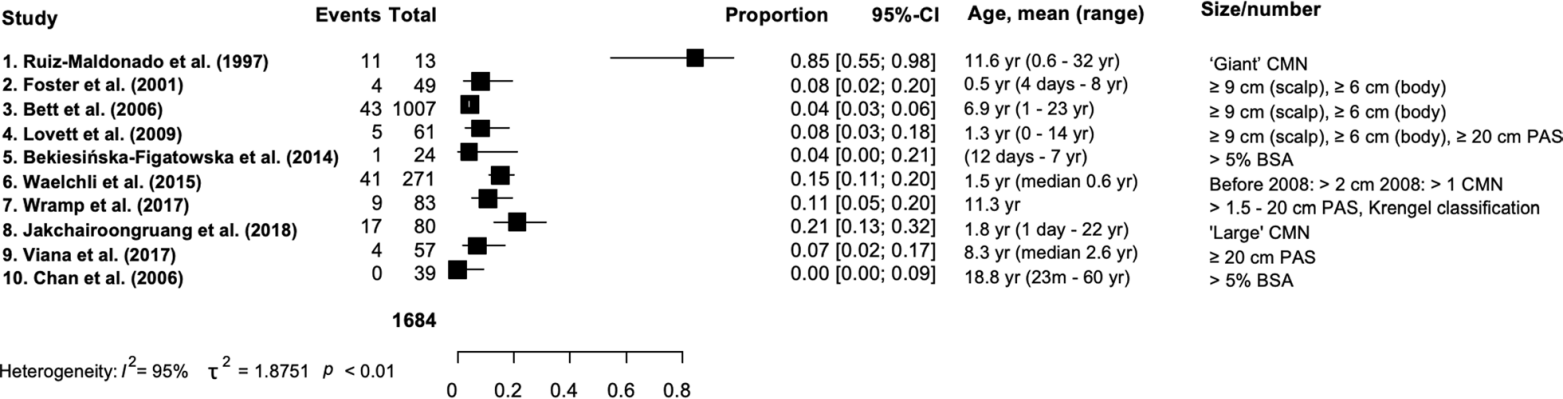
Informative forest plot for prevalence of neurological signs and symptoms in a general CMN population

**Fig. 4:**
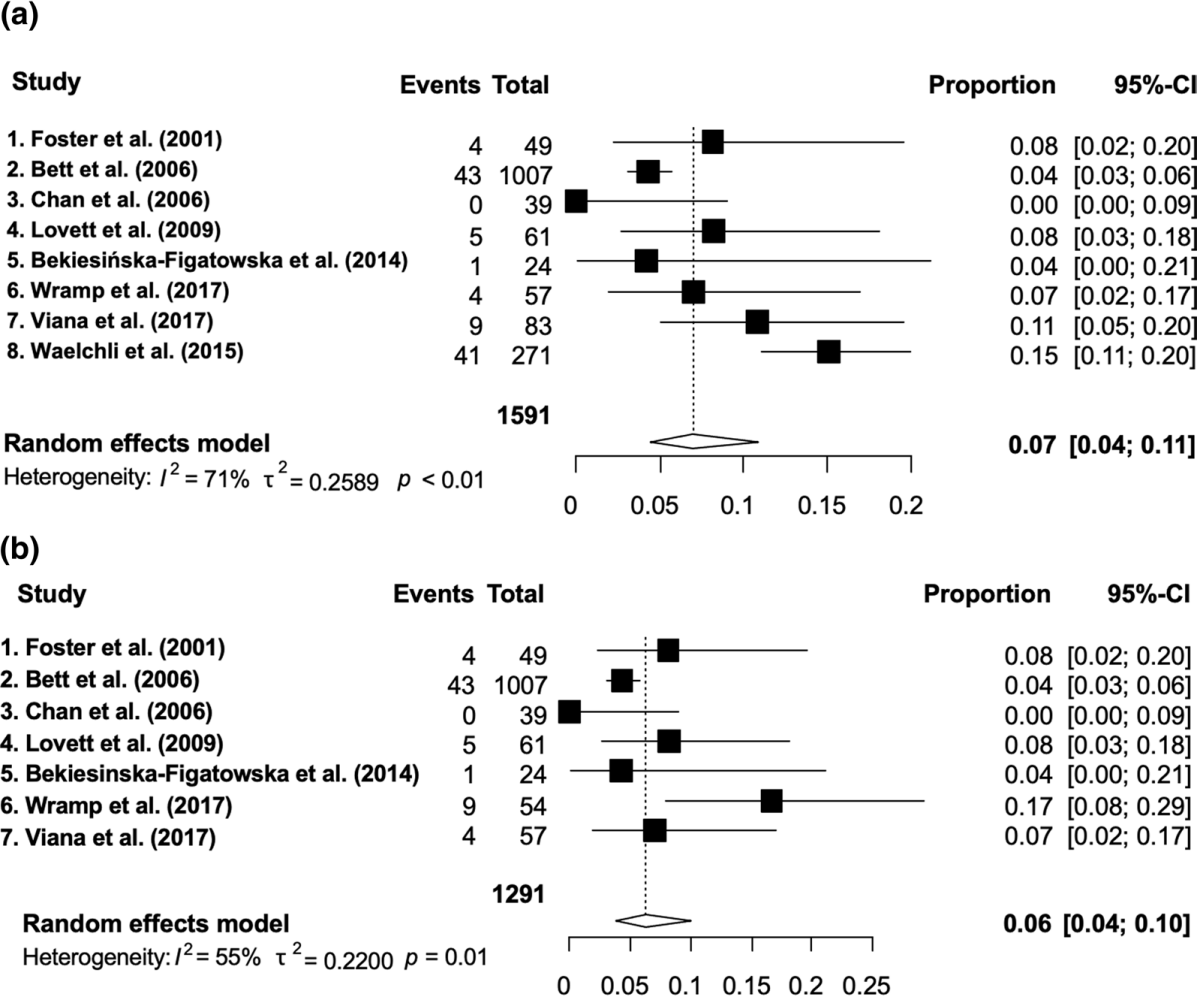
Forest plot: estimation of prevalence of neurological signs and symptoms. **a** Studies with all sizes of CMN and **b** patients with CMN size of at least > 6 cm in children or multiple medium CMN

Descriptive statistics of neurological signs and symptoms, MRI abnormalities and patient death are shown in Tables 2, 3 and 4 respectively. A meta-analysis could only be performed for neurological signs and symptoms prevalence and not for MRI abnormality, due to incomplete data. For the analysis, we excluded four studies that only reported on neurological signs and symptoms in patients with MRI abnormalities. Furthermore, we excluded two studies due to (1) much higher prevalence of neurological signs and symptoms (84.5%) [17]; this discrepancy may have resulted from a selection bias, although we could not ascertain this as the patient selection criteria for this study was unclear, and (2) a study design where patients that had undergone MRI imaging were selected from a large database, potentially causing a bias, as it is likely that MRI is performed in individuals in this database considered to be at higher risk of neurological involvement [18]. With our first analysis we found neurological signs and symptoms in 7.04% (CI 95% 4.47–10.93%) in the CMN population, with an I^2^ of 71% representing considerable statistical heterogeneity. Therefore, we performed a subgroup analysis of patients with similar characteristics: CMN size > 6 cm or > 20 cm in adults or multiple medium CMN. In this group we found neurological signs and symptoms in 6.26% (95% CI 3.85–10%) with moderate heterogeneity (I^2^ = 55%). The forest plots are shown in Figs. 3 and 4. The mean age of the patients in the different studies did not correlate with the proportion of patients with neurological signs and symptoms.

**Table 2:** Neurological signs and symptoms in CMN patients

References	Patient number^†^	Symptoms signs n (%)	Epilepsy/seizures n (%)	Neurodevelopmental delay n (%)	Symptoms of increased intracranial pressure/Hydrocephalus^‡^ n (%)	Other n
Group 1: General CMN group						
Ruiz-Maldonado et al. [17]	13	11/13 (85%)	1/13 (8%)	5/13 (38%) Impaired mental status (n = 4) Language retardation and brain immaturity (n = 1)	Unreported	Cephalalgia (headache) (n = 4) Pyramidal syndrome (n = 5) Dysesthesia in nevus (n = 4) Soft signs (n = 3) Cranial nerves dysfunction (n = 2) Hypoacusis (n = 1) Motor deficit (n = 1) Somniloquy (sleep-talking) (n = 1)
Foster et al. [9]	46	1/46 (2%) 4/49 (8%)^§^	Possible seizures, number unknown	1/46 (2%) Developmental delay (most notably in speech development)	Unreported	Generalized hypotonia (n = 1)
Bett et al. [48]	1008 (One patient with a Cafe au lait macula)	43/1008 (4%)	25/1008 (2%)	10/1008 (1%) Developmental delay (n = 7) Speech delay (n = 4)	28/1008 (3%) Hydrocephalus	Depression (n = 2) ADHD (n = 1) Autism (n = 1) Behaviour disorder (n = 1) Incontinence (n = 1) PTSD (n = 1) Strabismus (n = 1) Substance abuse (n = 1)
Chan et al. [25]	39	0/39 (0%)	0/39 (0%)	0/39 (0%)	0/39 (0%)	0
Lovett et al. [20]	61	5/61 (8%)	3/61 (5%)	1/61 (2%) Learning difficulties	1/61 (2%) Increased intracranial pressure	Behavioural problems (n = 1)
Bekiesińska-Figatowska et al. [47]	24	1/24 (4%)	1/24 (4%)	Unreported	Unreported	Unreported
Waelchli et al. [7]	271	41/271 (15%)	17/263 (6%)	41/271 (15%)	6/18 (33%)^¶^	Unreported
Viana et al. [56]	57	4/57 (7%)	Seizures (number unknown)	Number unreported Delay in neuropsychomotor development	Unreported	Unreported
Wramp et al. [24]	83	9/83 (11%)	1/83 (1%)	3/83 (4%)	2/83 (2%) Increased intracranial pressure	Locomotor impairment (n = 2) Deafness in left ear (n = 1) Difficulties concentrating (n = 1) Central sleep apnoea (n = 1) Hearing impairment in both ears (n = 1) Visual impairment (n = 1)
Jakchairoongruang et al. [18]	80	17/80 (21%)	Seizures (number unknown)	Unreported	≥ 5 Exact number unreported	Unreported
Group 2: Only reporting on neurological signs and symptoms in patients with MRI abnormalities						
Bittencourt et al. [23]	13	9/13 (69%)	5/13 (38%)	Unreported	4/13 (31%) Hydrocephalus	Decreased arm function (n = 1) Paraparesis (n = 1)
Ramaswamy et al. [19]	14	14/14 (100%)	7/14 (50%)	6/14 (43%) Delayed development (n = 5) Mild motor and cognitive delay (n = 1)	3/14 (14%) Hydrocephalus 1/14 (7%) Headache due to increased intracranial pressure	West syndrome (n = 1)
Price et al. [55]	12	5/12 (42%)	Unreported	Unreported	Unreported	Unreported
Qian et al. [21]	13	13/13 (100%)	8/13 (62%)	11/13 (85%) Cognitive impairment	9/13 (69%) Hydrocephalus on MRI 13/13 (100%) Acute or subacute headache and intracranial pressure 4/13 Ventriculoperitoneal shunt	Abduction nerve paralysis, hearing loss, vision loss (n = 1)

We identified two study groups. Ten studies reported on neurological signs and symptoms and MRI imaging in a general CMN population ‘general CMN group’ (2107 patients) and four studies reported on neurological signs and symptoms only in patients with MRI abnormalities ‘only reporting on neurological signs and symptoms in patients with MRI abnormalities’(232 patients)

ADHD, Attention Deficit/Hyperactivity Disorder; CMN, congenital melanocytic naevi; CNS, central nervous system; PTSD, Post-traumatic stress disorder

^†^This number represents the group of CMN patients where the proportion of symptoms and signs can be calculated. Patients that were excluded by the individual studies were included in our studies if information about neurological involvement was available about these excluded patients

^‡^Hydrocephalus is a radiological diagnosis and not a symptom, but sometimes it was classified as a symptom, however, it was not clear if the diagnosis hydrocephalus was accompanied with symptoms or signs

^§^Three patients were excluded because they already had symptoms before the study. A total of 4 patients of 49 had symptoms/signs

^¶^Six (33%) of 18 subjects with 'additional pathology' beside intraparenchymal melanocytosis had hydrocephalus on MRI, and 5/6 were asymptomatic

**Table 3:** MRI characteristics/abnormalities and location of melanocytosis

References	MRI		Location of MRI abnormalities		Others
	MRI performed n	MRI abnormalities n (%)	Leptomeningeal melanocytosis n (%)	Parenchymatous melanocytosis n (%)	Other findings than melanocytosis
Group 1: General CMN group					
Ruiz-Maldonado et al. [17]	13/13	7/13 (45%) No CNS melanocytosis	Not applicable	Not applicable	Ventricular system asymmetry (n = 4) Calcifications (n = 2) Large cisterna magna (n = 2) Cortical atrophy, loss of cortico subcortical volume (n = 1) Right frontotemporal subgaleal collection (n = 1)
Foster et al. [9]	42/46	14/42 (33%) (10 CNS melanocytosis)	2/10 (20%)	10/10 (100%)	Middle cranial fossa arachnoid cyst (n = 1) Chiari type 1 malformation (n = 1) Tethered spinal cord secondary to a filum terminale fibrolipoma (n = 1) Transient crescentic enhancement over the right parietal convexity (that was not evident on repeated examination seven months later) (n = 1)
Bett et al. [48]	Unreported	Unreported	Unreported	Unreported	Dandy-Walker complex (n = 5) Right hemimegalencephaly (n = 1) Cerebral cortical dysplasia (n = 1) Cerebral matrix haemorrhage (n = 1) Chiari malformation (n = 1) Choroid plexus tumour (n = 1) Encephalocraniocutaneous lipomatosis (n = 1) Tethered cord (n = 1) Unknown tumour (n = 1)
Chan et al. [25]	7/39 Head (n = 5) Spine (n = 2)	0/0 (0%)	Not applicable	Not applicable	Not applicable
Lovett et al. [20]	27/61 (and 1 CT and 1 myelogram)	7/27 (26%) (and 1 CT abnormality)	Unreported	Unreported	CT scan: 2 hyperdense foci (n = 1) Spinal cord MRI: Mega cisterna magna, increased amount of fluid in post fossa with hydromyelia from C4-T6 (n = 1) Brain MRI: arachnoid cyst (n = 1) Ventriculomegaly with haemorrhagic changes, VP shunt, diffuse enhancement of meninges, intraparenchymal hematoma (n = 1)
Bekiesińska-Figatowska et al. [47]	24/24	8/24 (33%) (CNS melanocytosis)	4/7 (57%)	7/7 (100%)	Neurofibromatosis type 1 (multiple multilevel roots neurofibromas on MRI) (n = 1)
Waelchli et al. [7]	271/271	46/271 (17%) (36 (13%) CNS melanocytosis)	3/36 (8%)	35/36 (97%)	Dandy-Walker malformation with hydrocephalus (n = 2) Lack of white matter bulk (n = 2) Larger ventricles (n = 2) Benign intradural tumour (n = 1) Choroid plexus papilloma (n = 1) Cortical thinning (n = 1) Extramedullary dural stranding (n = 1) Filum terminal lipoma (n = 1) Left frontal lobe meningioma (n = 1) Leptomeningeal disease (n = 1) Low volume inferior vermis (n = 1) Midline posterior fossa arachnoid cyst (n = 1) Right cerebellar astrocytoma (n = 1) Small right cerebellar hemisphere (n = 1) Spinal cord compression (n = 1) Venous angioma left cerebellar hemisphere (n = 1)
Viana et al. [56]	11/57	Unreported	Unreported	Unreported	Unreported
Wramp et al. [24]	36/83	4/36 (11%) (2 CNS melanocytosis)	Unreported	Unreported	Unreported
Jakchairoongruang et al. [18]	80/80	35/80 (41%) (33 CNS melanocytosis)	5/33 (15%)	33/33 (100%)	Periventricular grey matter heterotopia (n = 3) Dysmorphic cerebellar hemispheres (n = 2) Small left-side ventral pons (n = 2) Small pons and cerebellum (n = 2) Corpus callosum hypogenesis (n = 1) Inferior vermian hypoplasia (n = 1) Small right cerebellar hemisphere (n = 1) Right temporal lobe polymicrogyria (n = 1) Vermian hypoplasia (n = 1)
Group 2: Only reporting on neurological signs and symptoms in patients with MRI abnormalities					
Bittencourt et al. [23]	38/194	13/38 (34%) (CNS melanocytosis)	Unreported	Unreported	Dandy-Walker syndrome (n = 1)
Ramaswamy et al. [19]	14/14	14/14 (100%)	7/14 (54%) Diffuse leptomeningeal deposits	8/14 (62%)	Lower cervical benign spindle cell tumor (n = 1) Holocord arachnoid cyst (n = 1) Cervical/thoracic cyst (n = 1) Dorsal thoracic cyst (n = 1)
Price et al. [55]	Unreported	12 (CNS melanocytosis)	Unreported	Unreported	Unreported
Qian et al. [21]	13/13	13/13 (100%) (CNS melanocytosis)	13/13 (100%) Diffuse leptomeningeal deposits	Unreported	Leptomeningeal thickening (n = 13)

We identified two study groups. Ten studies reported on neurological signs and symptoms and MRI imaging in a general CMN population ‘general CMN group’ (2107 patients) and four studies reported on neurological signs and symptoms only in patients with MRI abnormalities ‘only reporting on neurological signs and symptoms in patients with MRI abnormalities’ (232 patients)

CMN, congenital melanocytic naevi; CNS, central nervous system

**Table 4:** Patients with CMN who died due to neurological complications

Total number of patients who died	34 (of a total of 2339 patients), excluding the studies that only reported neurological signs and symptoms in patients with MRI abnormalities: 24 (of a total of 2107 patients)
Age of neurological diagnosis	Mean: 5.86 years, median: 3 years, range: birth–27 years
Age of death	Mean: 7.44 years, median: 5.15 years, range: 0.7–28 years
Time between diagnosis and death	Mean: 1.43 years, median: 0.76 years, range: 0–4.4 years
Sex	Female: (n = 14), male: (n = 20)
Cause of death	Proliferating melanocytosis of the CNS (n = 19), malignant melanoma (n = 15)
Number of CMN	Multiple (n = 27), single (n = 0), unreported (n = 7)
Symptoms/signs	Neurodevelopmental delay (n = 9), seizures (n = 11), hydrocephalus/increased intracranial pressure (n = 28)

It was not possible to perform subgroup analysis in different CMN patient groups based on the age, size, number or locations of CMN groups, due to the lack of uniform definition, clinical heterogeneity or missing data. The quality of evidence regarding the prevalence of neurological signs and symptoms was very low according to the GRADE methodology due to heterogeneity, imprecision of estimates and high risk of bias. The summary of findings table is shown in Additional file 1: S3.

Out of 2339 patients included in the study, 576 received an MRI. When excluding the four studies that only reported on neurological signs and symptoms in patients with MRI abnormalities, 511/2107 (24%) patients had an MRI scan. MRI abnormalities were reported in 130 patients (25% of scans). The exact number of patients with MRI abnormalities but no neurological signs and symptoms was unknown, but at least 51 patients in this category were reported. At least 24 patients were reported with neurological signs and symptoms but without MRI abnormalities, but the exact number in this category was unknown as well. The correlation between the amount of CNS melanocytosis and the risk of neurological signs and symptoms or mortality is unclear [19, 20]. The patients with diffuse leptomeningeal melanocytosis had poor prognosis [21]. One study reported that isolated intraparenchymal melanocytosis was less associated with need for surgical intervention or mortality, compared with melanocytosis in combination with other MRI abnormalities [7].

The most frequently reported neurological signs and symptoms were seizures and neurodevelopmental delay, although the exact number is unclear. Melanocytosis, described as increased signal on T1-weighted images with sometimes concomitant decreased signal on T2-weighted images, was the most frequently reported MRI abnormality. The most frequently reported location of melanocytosis was in the brain parenchyma (Table 3). Hydrocephalus was frequently described, but not clearly defined. Enlargement of the ventricles of the brain on imaging studies is by definition hydrocephalus, although the clinical relevance of this finding depends on the underlying cause and the presence of symptoms. For instance, an obstruction to cerebrospinal fluid flow causing increased intracranial pressure requires treatment (i.e. shunt placement) while non-progressive enlargement of the ventricles of the brain without symptoms can be considered an incidental finding.

Death was reported for 34 patients (of the total 2339 patients), their details are found in Table 4 and Additional file 1: S4. Death was due to CNS malignant melanoma (15/34) or proliferating melanocytosis (19/34), a persisting proliferation of melanocytes, with rapid clinical deterioration leading to severe increased intracranial pressure and subsequent death [8, 22]. Additional file 1: S5 shows the central nervous system melanoma found in the included studies.

Six studies made recommendations concerning indications for routine MRI-screening [7, 19, 20, 23–25] and one study made recommendations for imaging techniques [18]. Five studies recommended routine MRI-screening in the ‘high-risk groups’ [7, 19, 20, 23, 24]. However, the definition of high-risk group differs between studies (Table 5). Another study argued that screening might not be cost-effective as the absolute risk of neurological involvement in larger CMN appeared to be low in their study [25].

**Table 5:** The various definitions for high-risk patients who are suggested to receive routine MRI-screening

References	Definitions
Bittencourt et al. [23]	Large CMN on the head, neck or over the dorsal spinal cord
Lovett et al. [20]	Large CMN on the head, neck or over the dorsal spinal cord or with multiple satellites
Ramaswamy et al. [19]	CMN on the head, neck or over the dorsal spinal cord
Waelchli et al. [7]	Children with two or more CMN at birth, independent of projected adult size or site of the largest CMN
Wramp et al. [24]	CMN of > 40 cm projected adult size or with > 20 satellites

## Discussion

This study provides an overview of neurological signs and symptoms and CNS MRI abnormalities in patients with CMN reported in the medical literature. We found a neurological signs and symptoms prevalence of 6.26% (95% CI 3.85–10%) in patients with a CMN > 6 cm or multiple medium CMN. A quarter of the performed MRIs (25%) found neurological abnormalities. Due to low quality of evidence, it is not possible to state the prevalence of these outcomes with certainty and to make an association between specific MRI abnormalities and neurological signs and symptoms. The increased risk of clinically relevant neurological abnormalities in patients with CMN can therefore not be estimated.

The most frequently reported neurological signs and symptoms were neurodevelopmental delay and seizures. The risk of these neurological signs and symptoms in CMN could not be estimated due to incomplete data or of poor definitions. An estimated 0.5–1% of the general pediatric population will experience at least one afebrile seizure [26–29]. The prevalence of neurodevelopment delay in patients with CMN could not be compared to the general paediatric population as clear definitions of neurodevelopment delay were generally missing in the CMN studies. One study performing routine MRI for all patients, showed seizures or neurodevelopmental delay in patients with CMN without MRI abnormalities [7]. However, these neurological signs and symptoms were milder and less frequent than neurological signs and symptoms in the group with MRI abnormalities, implicating an association between these neurological signs and symptoms and MRI abnormalities [7].

The most frequently reported MRI abnormalities were melanocytosis (described as increased signal on T1-weighted or decreased signal on T2-weighted MRI) and hydrocephalus. Increased intracranial pressure can be caused by obstruction of the ventricular system due to melanocytosis [22]. The severity and nature of the hydrocephalus was not well reported in the different studies. It is often unclear whether it refers to the radiological finding of enlarged ventricles (for which there are many causes, including diffuse brain atrophy) or the clinical syndrome of hydrocephalus due to an obstruction in cerebrospinal fluid flow causing raised intracranial pressure and requiring treatment.

Other MRI abnormalities besides melanocytosis and hydrocephalus were described as well (Table 3). On one hand, these MRI abnormalities may be considered as incidental findings, as incidental brain MRI findings are common in the paediatric population [30, 31]. On the other hand, these findings might be a part of the “CMN syndrome” i.e. the combination of cutaneous CMN with additional abnormalities [7, 32, 33]. For instance, Dandy-Walker syndrome was found in eight patients with CMN, an association documented in other articles [34–36]. It was difficult to determine what specific MRI abnormalities are associated with CMN.

We could not perform a subgroup analysis and estimate the risk of neurological involvement in different subgroups based on CMN locations, sizes, or number. It has been suggested that CMN location on the head, neck or spine should no longer be considered a risk factor for neurological involvement but rather a confounder for large or giant CMN [5, 7].

Studies have shown that the risk increases with the size of the largest CMN and the number of satellites [1, 3, 5, 37, 38]. We found a positive correlation between increased number of satellites and mortality. However, rare cases are described of individuals with no evident CMN and CNS melanocytosis [21, 39, 40]. These cases did not meet our inclusion criteria and might be underestimated in this study. Nonetheless, when mutations occur in the ectoderm in an early embryotic stage, mutations could affect both the CNS and large or multiple areas of the skin making larger CMN (> 20 cm PAS) or the multiple CMN the ‘high risk group’ for neurological involvement [32, 41].

In contrast to the older studies [1, 42, 43], recent studies show that the prognosis of symptomatic patients with CMN is not necessarily poor [5, 7, 19]. The proportion of deceased patients with CMN reported in these studies may be an overestimation as deceased patients may be better documented compared to asymptomatic patients. The majority of deceased patients had multiple cutaneous satellites as presented in the Additional file 1: S4. This is explained by the fact that patients with neurocutaneous melanocytosis frequently have multiple satellite lesions. However, having multiple satellite lesions is not necessarily associated with a poor prognosis.

### MRI-screening

It is of great importance to detect neurological signs and symptoms at an early stage to provide adequate management [7, 8]. Epilepsy in patients with CMN can be often effectively treated by antiepileptic drugs, and if resistant, by surgery [44]. Increased intracranial pressure can be treated with ventriculo-peritoneal shunting, and tumours can be resected [7, 21]. It is debated whether routine MRI is needed to adequately manage neurological complications in an early stage [7, 8, 20, 25].

Experts state that clinical management can be substantially adapted by routine MRI-screening [7, 20]. Furthermore, it can be used to establish prognosis and prepare families and clinicians for possible severe complications [20, 45]. Collecting data by routine MRI-screening may also benefit future understanding of this rare disease [18]. Five of the included studies recommended routine baseline MRI-screening in all high-risk patients with CMN. However, the definitions of high-risk were heterogeneous (Table 5). The value of repeat MRI-screening in asymptomatic patients is debated [7, 19, 20, 46]. When routine MRI-screening is performed, it is advised to perform screening in the first four months of life as myelinization of the CNS may obscure the melanocytic lesions [18, 19, 47].

Others argue that advantages do not outweigh the inconveniences of routine MRI-screening including extra costs, need for anaesthesia, false positives/negatives and uncertainties of the predictive value of routine MRI-screening compared with routine neurological examination [8, 25]. It is unclear how many patients benefited from routine MRI and if patients that received MRI only when neurological signs and symptoms appeared had poorer neurological outcomes.

A false negative MRI can lead to false sense of security and misdiagnosis of neurological problems. Four fatal cases from neurological complications in patients with a negative baseline MRI are reported in the literature [7, 20, 47, 48]. This can be explained by a false negative MRI analysis [7, 20] or by the fact that new lesions developed after the baseline MRI [47].

Routine MRI may also cause overdiagnosis of neurological involvement in patients with CMN. Incidental MRI findings are common in the general paediatric population [30, 31] as well as other aetiologies than melanin can cause increased signal on T1-weighted images such as lipid, protein, calcium, iron, copper, and manganese [49]. Moreover, MRI abnormalities are not necessarily associated with severe neurological complications. The exact proportion is unidentified, but we found at least 51 patients with MRI abnormalities without neurological signs and symptoms. Patients with a positive MRI who never develop complications, could be exposed to unnecessary, possibly invasive, interventions and may live with a fear of severe complications. As it is not clear how many patients actually benefit from routine MRI screening and as routine MRI screening may be a burden for the patient and their family, the Dutch multidisciplinary CMN guidelines do not recommend routine MRI screening unless there is any doubt about the presence of neurological signs and symptoms [8]. In addition, the Dutch guidelines recommend yearly neurological evaluation at least until the age of five in patients with multiple CMN [8].

The strength of this review is the systematic approach to provide an overview of the evidence gaps of the current literature. The limitation of this study is the clinical heterogeneity and the high risk of bias that hindered accurate statistical data synthesis. The article with the best methodological quality had a higher prevalence of neurological signs and symptoms (15%) than our point-estimate (7.04%) [7]. This can be explained by the prospective study design, which implied adequate neurological signs and symptoms reporting. Another possible explanation is that patients with neurological signs and symptoms were more likely to visit that expert centre. From 2008, only patients with multiple CMNs were included, which may have further raised the neurological signs and symptoms prevalence. We did not correct for age. Studies with younger patients are expected to have less cases with neurological signs and symptoms, as young patients can still develop neurological signs and symptoms later in life. However, this was not seen in the results, the mean age of the study groups did not correlate with neurological signs and symptoms prevalence. This may be explained by the high risk of bias but might also imply that neurological signs and symptoms mainly appear at a younger age. Relevant articles in other languages than English or Dutch could be missed, however, there was a global representation of studies. Large heterogeneity was found between different aspects of the included studies. Firstly, classification of CMN was reported in different ways, this hindered subgroup analysis of different phenotypes. Secondly, the inclusion criteria were different between studies. For instance, the minimum size of CMN differed. Lastly, the reporting of outcomes was heterogeneous. Some studies described a wide variety of outcomes ranging from death to very mild neurological signs and symptoms, while other studies limited their results to a few predefined outcomes [7]. Furthermore, clear definitions for specific neurological signs and symptoms were missing. Especially ‘neurodevelopmental delay’ could be interpreted in various ways.

The prevalence of outcomes could be an over- or underestimation of the actual risk. Reporting bias could cause an underestimation as some neurological signs and symptoms, or MRI abnormalities might not be well reported in the retrospectively reviewed medical records. Publication and selection bias may have caused an overestimation as people with CMNs without neurological complications are less likely to visit specialized medical research centres or be included in registries. The prevalence of MRI abnormalities found in the patients receiving MRI (26%) may be an overestimation, as it is likely that clinicians reserve MRI investigations for individuals they consider to be at high risk, especially in the older studies as MRI was not a commonly used diagnostic tool [1, 42, 43].

The rarity of larger CMN (> 20 cm PAS) makes it difficult to conduct research with large sample sizes. To gain high-level evidence regarding CMN, uniformity and standard reporting is needed. We recommend the use of the Krengel classification and the 6B classification [50, 51] for homogenous baseline characteristics and the CMN core outcome set for homogenous outcomes, i.e., a consensus-based agreed minimum set of outcomes that should be measured and reported in all clinical research and care of CMN [14, 52–54]. Neurological signs and symptoms are selected as core outcomes and the next step will be to find a measurement instrument to standardize reporting on neurological signs and symptoms in patients with CMN. Our overview can support such a project. Furthermore, our review provides the best available evidence that can be used to inform patients and therefore enables shared decision making.

## Conclusion

Based on current evidence, it is not possible to make high-level evidence recommendations regarding routine MRI-screening. The risk of severe neurological complications in patients with CMN is unclear as well as it is not clear how many patients actually benefited from routine MRI. Standardization in studies and a multi-centre prospective study are needed to improve knowledge on neurological involvement and to evaluate MRI-screening.

For now, every clinical centre should decide on its own policy and weigh the advantages and disadvantages of MRI-screening in high risk CMN. Nonetheless, an MRI is recommended at any age, when an individual develops new neurological signs and symptoms [7, 8].

## Supplementary Information

**Additional file 1**. S1. Search strategy; S2. Risk of bias assessment; S3. CMN patients with melanoma of the central nervous system; S4. CMN patients who died due to neurological involvement; S5. CMN patients with melanoma of the central nervous system.

## Data Availability

All data generated or analysed during this study are included in this published article and its additional files.

## Abbreviations

CMN: Congenital melanocytic naevi
CNS: Central nervous system
GRADE: Grading of recommendations assessment, development and evaluation
JBI: Joanna Briggs institute
MOOSE: Meta-analyses Of Observational Studies in Epidemiology
MRI: Magnetic resonance imaging
PAS: Projected adult size
