# Supplementary material for: Neurological signs, symptoms and MRI abnormalities in patients with congenital melanocytic naevi and evaluation of routine MRI-screening: systematic review and meta-analysis
Source: Orphanet J Rare Dis. 2022 Mar 2;17:95. doi: 10.1186/s13023-022-02234-8 (PMC8889704; doi:10.1186/s13023-022-02234-8)
Supplement: Supplementary file 1 — Additional file 1. S1. Search strategy; S2. Risk of bias assessment; S3. CMN patients with melanoma of the central nervous system; S4. CMN patients who died due to neurological involvement; S5. CMN patients with melanoma of the central nervous system. [file 13023_2022_2234_MOESM1_ESM.docx]

**Supplementary file 1: Search strategy**

PubMed

("Magnetic Resonance Imaging"[Mesh] OR "Neuroimaging"[Mesh] OR "Neurologic Manifestations"[Mesh] OR "Brain Diseases"[Mesh] OR "Neurodevelopmental Disorders"[Mesh] OR magnetic resonance imaging[tiab] OR MRI[tiab] OR neuroimaging[tiab] OR neurocutaneous melanocytosis[tiab] OR neurocutaneous melanocytosis[tiab] OR neurolog*[tiab] OR fits[tiab] OR epilep*[tiab] OR neuro*[tiab] OR seizure*[tiab] OR hydrocephalus[tiab] OR brain*[tiab] OR neurodevelop*[tiab]) AND ("Nevus"[Mesh] OR nevus[tiab]  OR nevi[tiab]  OR naevus[tiab]  OR naevi[tiab]  OR birthmark*[tiab]) AND ("congenital" [Subheading] OR congenital*[tiab]  OR bathing trunk*[tiab]  OR garment[tiab]  OR giant[tiab] OR tierfell*[tiab]  OR gigantic[tiab]  OR inborn[tiab]  OR hereditary[tiab]  OR newborn[tiab]) NOT ("Case Reports" [Publication Type] OR case report*[tiab])

EMBASE (Ovid):

Database(s): **Embase Classic+Embase**
Search Strategy:

| **#** | **Searches** |
| --- | --- |
| 1 | exp nuclear magnetic resonance imaging/ or exp neuroimaging/ or exp neurologic disease/ or exp brain disease/ or exp mental disease/ or (magnetic resonance imaging or MRI or neuroimaging or neurocutaneous melanocytosis or neurocutaneous melanocytosis or neurolog* or fits or epilep* or neuro* or seizure* or hydrocephalus or brain* or neurodevelop*).ti,ab,kw. |
| 2 | exp nevus/ or (nevus or nevi or naevus or naevi or birthmark*).ti,ab,kw. |
| 3 | (congenital* or bathing trunk* or garment or giant or tierfell* or gigantic or inborn or hereditary or newborn).ti,ab,kw. or cn.fs. |
| 4 | 1 and 2 and 3 |
| 5 | limit 4 to conference abstract status |
| 6 | 4 not 5 |
| 7 | case report/ or case report*.ti,ab,kw. |
| 8 | 6 not 7 |

Cochrane Library of Systematic Reviews and the Cochrane Central Register of Controlled Trials

ID Search Hits

#1 (magnetic resonance imaging or MRI or neuroimaging or neurocutaneous melanocytosis or neurocutaneous melanocytosis or neurolog* or fits or epilep* or neuro* or seizure* or hydrocephalus or brain* or neurodevelop*):ti,ab,kw

#2 (nevus or nevi or naevus or naevi or birthmark*):ti,ab,kw

#3 (congenital* or bathing trunk* or garment or giant or tierfell* or gigantic or inborn or hereditary or newborn):ti,ab,kw

**Supplementary file 2: Risk of bias assessment**


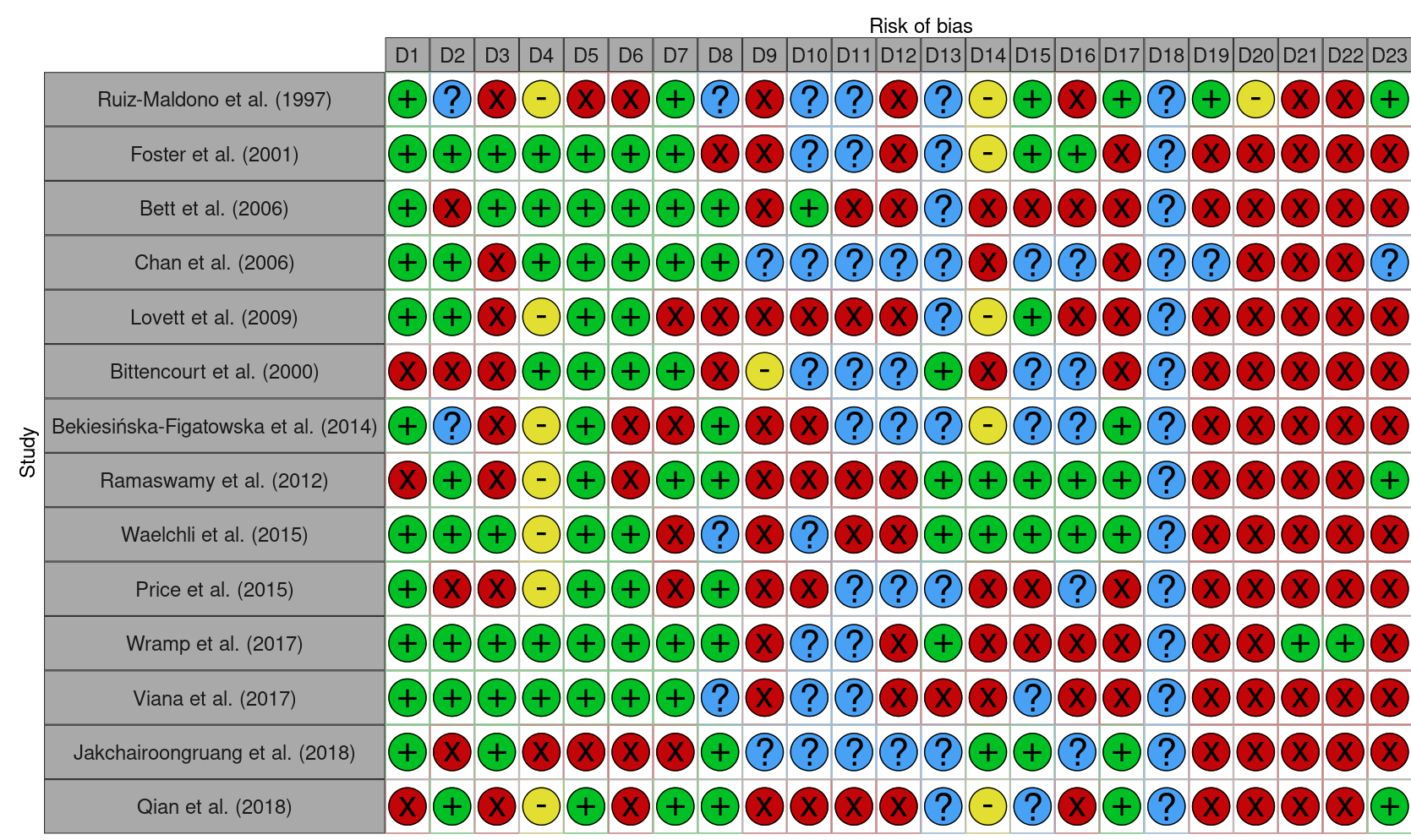


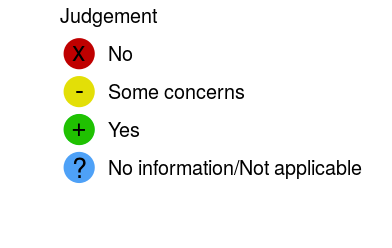
D1: 1. Was the sample representative of the target population?

D2: 2. Were the study participants recruited in a consecutive way?

D3: 3. Was the sample size adequate (n>40)?

D4: 4. Were the study subjects described in detail?

D5: 4a) Was the CMN size clearly defined?

D6: 4b) Was the number of CMN clearly defined?

D7: 4c) Was the age of subjects clearly defined?

D8: 5. Was the data analysis conducted with sufficient coverage of the identified sample?

D9: 6. Were objective, standard criteria used for the measurement of the condition?

D10: 6a) Was epilepsy clearly defined?

D11: 6b) Was hydrocephalus clearly defined?

D12: 6c) Was neurodevelopmental delay clearly defined?

D13: 6d) Is the melanoma in the central nervous system confirmed?

D14: 7. Was the condition measured reliably?

D15: 7a) Were the symptoms and signs identified by a clinician who is able to perform neurological examination?

D16: 7b) Did the authors describe the measurement instrument used for neurodevelopmental problems?

D17: 7c) Were MRI performed on all investigated subjects?

D18: 8. Was there appropriate statistical analysis?

D19: 9. Are important confounding factors for symptoms and signs described?

D20: 10. Were subpopulations identified using objective criteria?

D21: 10b) Was it possible to do a sub-group analysis of different size groups (according to the Krengel classifications)?

D22: 10d) Was it possible to do a sub-group analysis of different CMN number groups (according to the Krengel classifications)?

D23: 10f) Was it possible to do a sub-group analysis of different age groups?

**Supplementary file 3: CMN patients with melanoma of the central nervous system**

| **Outcome** | **Result** | **Subjects (studies)** | **Quality of evidence (GRADE)** |
| --- | --- | --- | --- |
| Prevalence of neurological symptoms and signs | 7.04 % (CI 95% 4.47% - 10.93%) in the whole group  6.26 % (95%CI: 3.85 - 10 %) patients with similar characteristics: a naevus > 6 cm or multiple medium naevi | 1591 (9 observational studies)  1291 (8 observetional studies) | ⨁◯◯◯  VERY LOW^a^ |
| Prevalence of MRI abnormalities | Not possible to estimate due to clinical heterogeneity, selection bias and missing deta |  | ⨁◯◯◯  VERY LOW^b^ |

a: very low quality of evidence due to heterogeneity, imprecision of estimate and high risk of bias.

b: not possible to estimate due to heterogeneity, selection bias and missing data.

**Supplementary file 4: CMN patients who died due to neurological involvement**

| **Article** | **Age (age diagnosis neurologic involvement/ age of**  **death)** | **Sex** | **Cause of death** | **CMN location/size/number of CMN** | **Signs and symptoms** | **MRI characteristics** |
| --- | --- | --- | --- | --- | --- | --- |
| **Bittencourt et al. (2000)** | ﻿78 months  ﻿† 87 months | ﻿Male | Proliferating melanocytosis of the CNS | ﻿Lumbosacral/  ≥ 20 cm/number unknown | Dandy-Walker ﻿syndrome | Positive for NCM |
|  | ﻿11 months (melanoma diagnosis)  † 13 months | Male | Cerebral melanoma | Back and trunk/  ≥ 20 cm/number unknown | ﻿Hydrocephalus | Positive for NCM |
|  | ﻿﻿20 months (melanoma diagnosis)  † 21 months | Female | Cerebral melanoma | ﻿Lumbosacral/  ≥ 20 cm/number unknown | ﻿﻿Decreased function of right arm | ﻿Not performed |
|  | ﻿36 months  † ﻿47 months | Female | Proliferating melanocytosis of the CNS | ﻿Trunk/  ≥ 20 cm/number unknown | ﻿Seizures,  Hydrocephalus | Positive for NCM |
| **Bett et al. (2006)** | 4 years  †4 years | Male | Meningeal melanoma | Bathing trunk nevus/  ≥ 20 cm/with satellites | Unreported | ﻿Hydrocephalus |
|  | 11 years  † 11 years | Male | Meningeal melanoma | Bathing trunk/  ≥ 20 cm/number of satellites unknown | Unreported | ﻿Hydrocephalus |
|  | 23 years  † 23 years | Male | Meningeal melanoma | Back /  ≥ 20 cm/  with satellites | Unreported | ﻿Hydrocephalus |
|  | 27 years  † 28 years | Female | Meningeal melanoma | Bathing trunk/  ≥ 20 cm/  with satellites | Unreported | Hydrocephalus (developed at age 27 years) |
|  | 9 months  † 10 months | Male | Proliferating melanocytosis of the CNS | ﻿Bathing trunk/  ≥ 20 cm/ with satellites | Unreported | ﻿Hydrocephalus  Leptomeningeal melanocytosis |
|  | Infancy  † 7 years | Male | Proliferating melanocytosis of the CNS | Bathing trunk/  ≥ 20 cm/ satellites number unknown | Seizures,  Neurodevelopmental delay | ﻿Dandy-Walker complex  Hydrocephalus as infant |
|  | 3 years  † 3 years | Female | Proliferating melanocytosis of the CNS | Bathing trunk/  ≥ 20 cm/ with satellites |  | ﻿Hydrocephalus |
|  | 3 years  † 4 years | Female | Proliferating melanocytosis of the CNS | Bathing trunk  ≥ 20 cm/ with satellites | Seizures | ﻿Normal MRI at birth  Later: hydrocephalus |
|  | 3 years  † 9 years | Female | Proliferating melanocytosis of the CNS | Back/  ≥ 20 cm/ with satellites | Seizures (at age 3),  Neurodevelopmental delay | ﻿Hydrocephalus |
|  | Unknows  † 11 years | Male | Proliferating melanocytosis of the CNS | Torso/  ≥ 20 cm/ with satellites | Unreported | ﻿Hydrocephalus |
|  | ﻿﻿14 months  †28 months | Female | Cerebral melanoma | Medium, multiple CMN, 30 satellites | Unreported | ﻿Hydrocephalus (only at age 14 months)  Positive for NCM (24 months with brain melanoma) |
|  | 1 months  † 9 months | Female | Proliferating melanocytosis of the CNS | Medium multiple CMN, many satellites | Unreported | ﻿Hydrocephalus (at 1 months)  Dandy-Walker complex |
|  | ﻿9 months  † 18 months | Female | Proliferating melanocytosis of the CNS | Medium multiple CMN, many satellites | ﻿Seizures | ﻿Hydrocephalus  Dandy-Walker complex |
|  | 4 months  † 37 months | Male | Proliferating melanocytosis of the CNS | Medium multiple CMN, many satellites | Seizures | ﻿Hydrocephalus, Positive for NCM |
|  | 2 years  † 2 years | Female | Proliferating melanocytosis of the CNS | Medium multiple CMN, many satellites | Unreported | ﻿Hydrocephalus, Positive for NCM |
|  | 5 years  † 8 years | Male | Proliferating melanocytosis of the CNS | Medium multiple CMN, many satellites | Unreported | ﻿Hydrocephalus, Positive for NCM |
|  | < 8 years  † 8 years | Male | Proliferating melanocytosis of the CNS | Medium multiple CMN, many satellites | Unreported | ﻿Hydrocephalus |
| **Lovett et al. (2009)** | 10 months  † 13 months | Female | Proliferating melanocytosis of the CNS  Uncontrollable intracranial pressure | Trunk anterior and posterior/  > 20 cm/multiple satellites (>20) | Initially presented with vomiting and signs of increased intra- cranial pressure.  Deteriorating mental status  Seizures (probably due to hemorrhage caused by V-P shunt)  Encephalopathy  Ophthalmology: peripapillary pigmentation | Positive for NCM  Size of ventricles diffuse enhancement of meninges |
| **Waelchi et al. (2015)** | Unknown | Male | Cerebral melanoma | Multiple CMN (exact number and size unknown) | ﻿Neurodevelopmental delay by time of first MRI | ﻿Posterior fossa malignant melanoma |
|  | Unknown | Female | Proliferating melanocytosis of the CNS | Multiple medium CMN/ largest lesion projected adult size < 5 cm/ > ﻿200 nevi | Seizures,  Neurodevelopmental delay (moderate global delay)  No symptoms at the time of first MRI | ﻿Diffuse leptomeningeal melanocytosis and Dandy Walker Malformations with hydrocephalus at 2 weeks.  Congenital leptomeningeal disease at 13 days.  At 6 months diffuse leptomeningeal melanoma |
|  | Unknown | Female |  | Location unknown / CMN > 60 projected adult size/ 20-50 satellites | Neurodevelopmental delay (mild) by time of first MRI | ﻿Intraparenchymal melanocytosis and subsequent diffuse leptomeningeal melanoma Hydrocephalus |
|  | Unreported | Unreported | ﻿Primary CNS melanoma | Unreported | Unreported | ﻿Normal initial MRI scan |
| **Wramp et al. (2017)** | 5.4 years  † 5.8 years | Male | Cerebral melanoma (NRAS-Q61R mutation) | Lumbar spine/ 20–30 cm projected adult size/ few satellites (<20) | Symptoms of increased intracranial pressure | Positive for NCM |
|  | ﻿9 years  † 9 years  Died a few weeks after the diagnosis | Female | ﻿Metastasized cerebral melanoma | ﻿Location unknows/ >60 cm projected adult size/ >50 satellites | ﻿Asymptomatic at time of research, developed symptoms shortly before the diagnosis | Positive for NCM |
| **Ramaswamy et al. (2012)** | 15 months  † 19 months | Male | ﻿No leptomeningeal  at 15 months  biopsy performed, but spinal lesion benign. CSF negative for malignancy. | Unreported | ﻿Epilepsy, focal  at 15 months,  ﻿Neurodevelopmental delay | ﻿Diffuse leptomeningeal deposits of the brain and spine, ﻿lower cervical benign spindle cell tumor |
|  | 13 months  † 23 moths | Male | ﻿Leptomeningeal melanoma | Unreported | ﻿Epilepsy, focal  age 13 months, Neurodevelopmental delay | Diffuse leptomeningeal deposits of the brain and spine |
|  | Birth  † 4.5 years | Male | ﻿Right cerebellar melanoma, skin negative for malignancy | Unreported | ﻿Hydrocephalus,  Neurodevelopmental delay,  ﻿Normal development until hydrocephalus developed | Diffuse leptomeningeal deposits of the brain and spine |
|  | 9 years  † 9 years | Male | ﻿CNS and skin negative for malignancy. No leptomeningeal biopsy done | Unreported | ﻿Hydrocephalus,  ﻿Normal development | Diffuse leptomeningeal deposits of the brain and spine |
|  | 8 years  † 10.5 years | Male | ﻿Left gyrus rectus  biopsy: leptomeningeal melanoma | Unreported | ﻿Epilepsy, focal  age 8 years,  Neurodevelopmental delay,  Normal development until age 8 then progressive decline | Diffuse leptomeningeal deposits of the brain, normal spine MRI |
|  | 25 years  † 28 years | Male | ﻿Right frontal  melanoma,  CSF positive for malignancy | Unreported | ﻿Headaches due  to increased intracranial  pressure,  Normal development | Diffuse leptomeningeal deposits, ﻿Left temporal and ﻿mesial temporal, right frontal melanoma |

†: death, CMN: congenital melanocytic naevi, CNS: central nervous system, NCM: neurocutaneous melanocytosis,

**Supplementary file 5: CMN patients with melanoma of the central nervous system**

| **Author** | **Melanoma in CNS** |
| --- | --- |
| **Group 1: general CMN group** | |
| **Ruiz-Maldonado et al. (1997)** | Unreported |
| **Foster et al. (2001)** | 1/49 (one of the excluded patients) |
| **Bett et al. (2006)** | 6/1008 |
| **Chan et al. (2006)** | 0/39 |
| **Lovett et al. (2009)** | 0/61 |
| **Bekiesinska-Figatowska al. (2014)** | 1/24 (primary site unknown) |
| **Price et al. (2015)** | 1/45 (primary site unknown) |
| **Waelchi et al. (2015)** | 4/271 |
| **Viana et al (2017)** | 1/57 (primary site unknown) |
| **Wramp et al. (2017)** | 2/83 (primary site unknown (n=1)) |
| **Jakchairoongruanget al. (2018)** | Unreported |
| **Group 2:** **only reporting on NS&S in patients with MRI abnormalities** | |
| **Bittencourt et al. (2000)** | 2/194 (primary site unknown n=1) |
| **Ramaswamy et al. (2012)** | 4/14 |
| **Viana et al (2017)** | 1/57 (primary site unknown) |
| **Qian et al. (2018)** | 4/13 |
| **Total** | **22/1937** |
